# Susceptibility of Four Species of Aphids in Wheat to Seven Insecticides and Its Relationship to Detoxifying Enzymes

**DOI:** 10.3389/fphys.2020.623612

**Published:** 2021-01-18

**Authors:** Peipan Gong, Defeng Chen, Chao Wang, Mengyi Li, Xinan Li, Yunhui Zhang, Xiangrui Li, Xun Zhu

**Affiliations:** ^1^State Key Laboratory for Biology of Plant Diseases and Insect Pests, Institute of Plant Protection, Chinese Academy of Agricultural Sciences, Beijing, China; ^2^College of Plant Protection, Shenyang Agricultural University, Shenyang, China; ^3^Key Laboratory of Biology and Genetic Improvement of Horticultural Crops, Beijing Vegetable Research Center, Ministry of Agriculture, Beijing, China; ^4^Beijing Key Laboratory of Vegetable Germplasm Improvement, Beijing Academy of Agriculture and Forestry Sciences, Beijing, China

**Keywords:** imidacloprid, cytochrome P450 monooxygenase, synergist, toxicity difference, wheat aphids

## Abstract

*Sitobion avenae* (Fabricius), *Rhopalosiphum padi* (Linnaeus), *Schizaphis graminum* (Rondani), and *Metopolophium dirhodum* (Walker) (Hemiptera: Aphididae) are important pests of wheat and other cereals worldwide. In this study, the susceptibilities of four wheat aphid species to seven insecticides were assessed. Furthermore, the activities of carboxylesterase (CarE), glutathione S-transferase (GSTs), and cytochrome P450 monooxygenase (P450s) were determined in imidacloprid treated and untreated aphids. The results showed that the susceptibilities of four wheat aphid species to tested insecticides are different and *M. dirhodum* has shown higher tolerance to most insecticides. Relatively higher CarE and GST activities were observed in *M. dirhodum*, and P450s activities increased significantly in response to imidacloprid treatment. Moreover, susceptibility to imidacloprid were increased by the oxidase inhibitor piperonyl butoxide in *M. dirhodum* (20-fold). The results we have obtained imply that P450s may play an important role in imidacloprid metabolic process in *M. dirhodum.* We suggest that a highly species-specific approach is essential for managing *M. dirhodum*.

## Introduction

The English grain aphid *Sitobion avenae*, bird cherry-oat aphid *Rhopalosiphum padi*, greenbug aphid *Schizaphis graminum*, and *Metopolophium dirhodum* (Homoptera: Aphididae) are important pests of wheat and other cereals worldwide (Zhang et al., 2019). According to National Bureau of Statistic wheat was cultivated on approximately 25.0 million hectares of agricultural land, of which nearly15 million hectares are infested with cereal aphids, resulting in immeasurable yield losses annually in China (Yan et al., 2016; Gong et al., 2020). *Sitobion avenae* and *R. padi* are problematic pest aphid species for diverse wheat cultivars grown in various regions (Lu and Gao, 2009; Hu et al., 2016). *Metopolophium dirhodum* is the most abundant aphid species on cereals in the continental climate of central Europe (Honek et al., 2018). Wheat aphids cause damage by directly feeding on plants and by vectoring multiple plant pathogenic viruses (Lu et al., 2016). Additionally, these pests feed on wheat in a mixed-population with similar environmental conditions (Hu et al., 2015), but these wheat aphids have subtle different characteristics, such as host plant range and adaptability (Denno et al., 2000; Gianoli, 2000; Honek et al., 2006; Sun et al., 2009).

The management of wheat aphids relies primarily on the application of insecticides. The organophosphate (OP) insecticides parathion and disulfoton were commonly used for controlling *S. graminum* in the 1990s (Gao and Zhu, 2000). Pyrethroid insecticides were increasingly used for aphids’ management because of their efficient contact activity and environment friendly toxicity profile after many pests developed serious resistance to OP insecticides (Chen et al., 2017; Mackenzie et al., 2018). Neonicotinoids represent a fourth insecticide class after OPs, carbamates and pyrethroids. Imidacloprid, the first commercialized insecticide of this class with high effectiveness against several sucking pests (Tomizawa and Casida, 2003; Lu et al., 2016), and it has been used as a seed treatment for controlling grain aphids in recent years (Miao et al., 2014). However, concerning the environmental impacts and resistance, the application of botanical insecticides such as abamectin, matrine and rotenone has been augmented (Dias et al., 2019; Jesser et al., 2020). New types of insecticides provide more choices for pest management, but the effectiveness needs further studies.

While chemical control is an effective method for managing pests, the wide and intensive use of synthetic insecticides has resulted in resistance to many major insecticide classes in field populations of many pests, including aphids (Zhang et al., 2017). One of the major mechanisms involves the increased activities of esterase, glutathione S-transferase (GSTs), and cytochrome P450 monooxygenase (P450s) (Zhang et al., 2016b). Previous studies indicated that elevated carboxylesterase (CarE) levels contributed to the resistance to OP and carbamate insecticides in *S. graminum* (Ono et al., 1999) and *R. padi* (Chen et al., 2007). The increased production of these enzymes, especially P450s have been detected in vast insecticide-resistant pests, such as *Musca domestica* (Zhang et al., 2010; Feng et al., 2018), *Nilaparvata lugens* (Mao et al., 2019; Jin et al., 2019), *Ceratitis capitata* (Arouri et al., 2015), *Myzus persicae* (Panini et al., 2015), and *Helicoverpa armigera* (Wang et al., 2019). Moreover, enhanced P450s activities have contributed to neonicotinoid resistance were reported in *N. lugens* (Bass et al., 2011), *Drosophila melanogaster* (Harrop et al., 2018), and *R. padi* (Wang et al., 2018).

We found that the field occurrence of *M. dirhodum* is more serious than *S. avenae* and *R. padi* in the areas such as Beijing and Hebei province where with imidacloprid seed treatment, according to us investigate result in past several years (Li et al., 2019). An earlier investigation revealed that the tissue-specific constitutive overexpression of P450s confers tolerance to imidacloprid in *Rhynchophorus ferrugineus* (Antony et al., 2019). To uncover the potential mechanism of insensitivity of *M. dirhodum* to imidacloprid, the main detoxifying enzymes (CarE, GSTs and P450s) activities in four wheat aphids were analyzed, focusing on imidacloprid treated and untreated aphids. The effects of three synergists piperonyl butoxide (PBO), triphenyl phosphate (TPP), and diethyl maleate (DEM) on imidacloprid against four wheat aphids were also detected. Furthermore, the susceptibilities of four wheat aphids to the other six frequently used insecticides, including thiamethoxam, avermectin, beta-cypermethrin, omethoate, matrine, and rotenone belong to class of neonicotinoid, macrolide, pyrethroid, OP and botanical were determined, following an application of leaf dipping method.

## Materials and Methods

### Insect Populations

The population of *Sitobion avenae*, *Rhopalosiphum padi*, *Schizaphis graminum*, and *Metopolophium dirhodum* with no exposure to insecticides since collected from Langfang (Hebei, China) in 2013 (N39°30’29”, E 116°36’09”) and reared on wheat seedlings under standard conditions (20 ± 1°C and 60 ± 10% relative humidity with a 16-h light:8-h dark photoperiod).

### Pesticides, Synergists, and Other Chemicals

The insecticides used for bioassays included imidacloprid (98% purity; Ningbo Sanjiang Yinong Chemical Co., Ltd., China), thiamethoxam (98% purity; Zhejiang Heben Technology Co., Ltd., China), beta-cypermethrin (97% purity; Jiangsu Yangnong Chemical Co., Ltd., China), omethoate (40% emulsifiable concentrate; Hebei Xinxing Chemical Co., Ltd., China), matrine (40% purity; Nantong Shenyu Green Pharmaceutical Co., Ltd., China), rotenone (40% purity; Inner Mongolia Qingyuanbao Biological Technology Co., Ltd., China), and avermectin (92% purity; Shijiazhuang Shuguang Pharmaceutical Raw Material Medicine Co., Ltd., China). Synergists piperonyl butoxide (PBO; reagent grade), triphenyl phosphate (TPP; reagent grade), and diethyl maleate (DEM; reagent grade) were purchased from Sigma-Aldrich Shanghai Trading Co., Ltd., United States.

Fast blue B salt, α-naphthyl acetate (α-NA), eserine, reduced glutathione (GSH), coomassie brilliant blue G250, 1-chloro-2,4-dinitrochlorobenzene (CDNB), phenylmethylsulfonyl fluoride (PMSF), n-phenylthiourea (PTU), albumin bovine (BSA), ethylenediaminetetraacetic acid (EDTA), DL-dithiothreitol (DTT) were purchased from Sigma-Aldrich Shanghai Trading Co., Ltd., United States.

### Bioassay

A leaf-based insecticide bioassay method was performed as previously described (Zuo et al., 2016). Serial dilutions of the active ingredients from the tested insecticides were prepared using 0.1% Tween-80 in water. Wheat leaves containing animate apterous aphids were dipped in the insecticide dilutions for 3 s. Then, the leaves were removed from the solution, and residual droplets on the leaves were adsorbed with clean, dry filter paper. Three replicates of at least 30 aphids were used for each insecticide concentration, and 5 or 6 serial concentrations were used for each insecticide. Leaves dipped in 0.1% Tween-80 were used as a control. The aphids were maintained at 20 ± 1°C and 60 ± 10% relative humidity with a 16-h light/8-h dark photoperiod after treatment. Mortality was assessed after 24 h using a stereomicroscope and the mortality of control in every experiment request lower than 10%. Aphids were scored alive at least one leg can move after being touched with an anatomical needle. The limitation of this whole-body immersion method is the penetration of the compounds would be higher because acetone would help active ingredients to penetrate better in the cuticle.

### Enzyme Assays

The activities of enzymes were measured as previously described (Lu et al., 2013) with some modifications. Batches of approximately 40 apterous adult aphids of each species were manually homogenized in 200 μL ice-cold 0.1 M sodium phosphate buffer (pH 7.6) containing 1 mM EDTA, 1 mM DTT, 1 mM PTU, 1 mM PMSF, and 20% glycerol. Homogenates were then centrifuged at 4°C, 12,000 g for 15 min in microcentrifuge (Thermo Fisher, Germany). The supernatant was collected for CarE, GSTs, and P450s activities analyzed. Three replicates were conducted for each aphid species.

Carboxylesterase activity was determined using the α-naphthyl acetate (α-NA) as described earlier with some modifications (Tang et al., 2017). The total reaction volume of 250 μL per well of a 96-well microplate contained 150 μL reaction mix (contained 107 mM α-NA, 107 mM eserine and 0.01 g mL^–1^ Fast blue B salt), 50 μL of PBS and 50 μL of the enzyme source. Absorbance was measured at 600 nm and 30°C using the kinetic model for 5 min continuously in a microplate reader (FlexStation 3, United States).

The activity of GSTs was determined according to a slightly modified published method involving CDNB (Habig et al., 1974). Briefly, a 300 μL reaction mixture comprising 100 μL CDNB (1.2 mM) substrate solution, 100 μL GSH (6 mM), and 100 μL diluted enzyme solution was prepared, after which the absorbance was measured at 340 nm using the kinetic model for 10 min.

The monooxygenase enzyme activity of four aphids were measured by an insect function oxidase ELISA kit (Huabaitai Biotechnology Corporation, Beijing, China) as previously described (Cui et al., 2018) with slight modifications. The enzyme sources were transferred to microlon ELISA plates according to the manufacturer’s instructions, after which the absorbance at 450 nm was measured and the concentrations were calculated based on a standard curve (y = 0.0043x + 0.0389, *R*^2^ = 0.9973), the results are expressed as IU/L.

The protein contents of the enzyme solutions were determined by the Bradford method. A serial albumin bovine (BSA) concentration solutions and samples were measured together and the protein content of samples were calculated by a standard curve based on BSA solutions. Three replicates of 10 μL diluted enzyme solutions were mixed with 200 μL protein assay dye reagent. After a 5 min incubation, the absorbance at 595 nm was measured.

### Sample Collection for Inducible Enzyme Assays

Wheat seedlings (about 15 cm high) were immersed in imidacloprid solutions for 5 min, the concentrations were LC_50_ values that 62.21 mg L^–1^ for *R. padi*, 6.79 mg L^–1^ for *S. avenae*, 41.11 mg L^–1^ for *S. graminum* and 261.91 mg L^–1^ for *M. dirhodum*, respectively, then selected to petri dishes containing moistened filter paper after air-dried at room temperature for 30 min. The aphids still live after 24 h were collected for enzyme activities analysis, 120 aphids were collected for each aphid species.

### Synergism Bioassays

The effects of three synergists PBO, TPP, and DEM on imidacloprid against four wheat aphids were tested. Stocks of insecticides with synergists were prepared and adjusted to final concentrations by serial dilution with distilled water containing 0.1% (v/v) Tween-80 for the bioassays. The highest concentration of PBO, DEF and DEM used were 625 mg/L and the mortality as low as control (<10%). The bioassay method was performed as described in section “Bioassay.”

### Statistical Analysis

The mortality data were corrected based on the control mortality with Abbott’s formula. The LC_50_ values, 95% confidence intervals and slopes were calculated (Data Processing System software, v.7.05). Toxicity difference ratio (TDR) indicates the tolerance difference among pests. The TDR of tested insecticides to *M. dirhodum*, *S. graminum*, *R. padi*, and *S. avenae* were calculated based on the LC_50_ values. Differences in the enzyme activities among aphid species were analyzed using one-way ANOVA followed by Tukey’s multiple comparisons test and Student’s *t*-test was used to separate the means between control and imidacloprid treatments (*p* < 0.05) (GraphPad Prism, v.8.0.1).

## Results

### Bioassay

The result showed that four wheat aphids have different susceptibilities to the tested insecticides. All tested insecticides showed lowest toxicity to *M. dirhodum* and the toxicity difference ratio (TDR) were greater than 2.0, except matrine. Imidacloprid and avermectin showed highest toxicity to *S. avenae*, and the TDR were ranged from 0.10 to 4.02, and 0.39 to 1.15; thiamethoxam has highest toxicity to *S. graminum*, the TDR was ranged from 0.49 to 11.81; omethoate, beta-cypermethrin, matrine, and rotenone showed highest toxicity to *R. padi*. The toxicity of avermectin to four aphid species is roughly comparable with LC_50_ ranged from 3.92 to 11.48 mg L^–1^ (Table 1).

**TABLE 1:** The toxicity different ratio of seven insecticides to four aphid species in wheat.

Insecticides	Species	*N*^*a*^	Slope ± SE	*P-*value	LC_50_ (95% CI)^*b*^ mg/L	TDR^*c*^
Imidacloprid	*R. padi*	884	0.76 ± 0.19	0.0289	65.21(27.80−152.99)	1.00
	*S. avenae*	481	1.11 ± 0.08	0.0054	6.79(4.81−9.59)	0.10
	*S. graminum*	873	0.50 ± 0.08	0.0088	41.11(23.84−70.89)	0.63
	*M. dirhodum*	887	0.83 ± 0.07	0.0013	261.91(177.09−387.35)	4.02
Thiamethoxam	*R. padi*	932	0.93 ± 0.15	0.0079	37.01(21.50−63.72)	1.00
	*S. avenae*	393	0.91 ± 0.11	0.0042	125.47(72.34−217.62)	3.39
	*S. graminum*	429	0.80 ± 0.22	0.0343	17.96(5.59−57.63)	0.49
	*M. dirhodum*	812	1.38 ± 0.22	0.0078	436.97(195.03−979.07)	11.81
Omethoate	*R. padi*	603	1.07 ± 0.22	0.0157	12.87(6.20−26.69)	1.00
	*S. avenae*	323	1.32 ± 0.13	0.0020	21.07(16.63−32.58)	1.64
	*S. graminum*	325	0.52 ± 0.03	0.0019	76.09(64.95−89.14)	5.91
	*M. dirhodum*	812	1.25 ± 0.26	0.0408	133.73(59.07−302.721)	10.39
Beta-cypermethrin	*R. padi*	693	0.65 ± 0.06	0.0608	4.94(2.77−8.83)	1.00
	*S. avenae*	533	0.57 ± 0.07	0.0035	19.67(11.57−33.46)	3.98
	*S. graminum*	856	0.81 ± 0.05	0.0006	26.13(19.51−35.00)	5.29
	*M. dirhodum*	925	0.83 ± 0.04	0.0002	30.04(24.47−36.89)	6.08
Avermectin	*R. padi*	279	0.46 ± 0.08	0.0299	10.00(3.74−26.73)	1.00
	*S. avenae*	920	0.88 ± 0.14	0.0082	3.92(1.92−8.02)	0.39
	*S. graminum*	780	0.81 ± 0.10	0.0044	7.52(3.94−14.34)	0.75
	*M. dirhodum*	723	1.32 ± 0.13	0.0018	11.48(7.33−17.98)	1.15
Matrine	*R. padi*	647	1.36 ± 0.08	0.0005	8.17(6.20−10.77)	1.00
	*S. avenae*	701	1.32 ± 0.11	0.0012	22.62(16.60−30.81)	2.77
	*S. graminum*	758	1.31 ± 0.21	0.0079	15.53(8.81−27.36)	1.90
	*M. dirhodum*	714	1.50 ± 0.21	0.0186	20.12(11.79−34.36)	2.46
Rotenone	*R. padi*	785	1.39 ± 0.23	0.0085	8.71(4.36−17.39)	1.00
	*S. avenae*	771	0.83 ± 0.17	0.0084	39.61(11.36−138.08)	4.55
	*S. graminum*	1205	1.19 ± 0.28	0.0242	29.53(11.68−74.65)	3.39
	*M. dirhodum*	915	0.93 ± 0.021	0.0001	118.03(105.04−132.64)	13.55

*^*a*^The number of aphids tested.*

*^*b*^Median lethal concentration (LC_50_), 95% confidence interval (95% CI).*

*^*c*^The toxicity difference of the insecticide between four aphid species based on LC_50_ values (other three aphid species LC_50_/R. padi LC_50_).*

### Enzyme Activity

**FIGURE 1:**
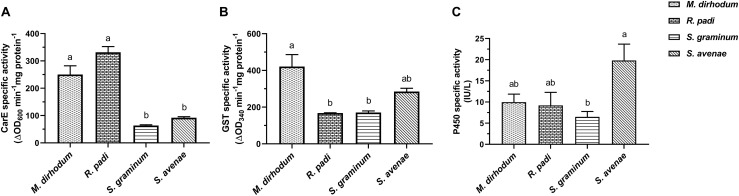
Specific activities of carboxylesterase (CarE, **A**), glutathione S-transferase (GSTs, **B**), and cytochrome P450 monooxygenase (P450s, **C**) in four aphid species in wheat. Data presented as the mean ± SEM (*n* = 3). Different letters on the bars indicate that the means are significantly different among the enzyme activities of aphid species using one-way ANOVA followed by Tukey’s multiple comparisons test (*p* < 0.05).

To find out what caused the differential susceptibility to insecticide among the four wheat aphids, and clarify the reasons of high insecticide tolerance in *M. dirhodum*, especially to imidacloprid, we determined the CarE, GSTs, and P450s activities. The data showed that CarE actives in *M. dirhodum* and *R. padi* were significantly higher than that in *S. graminum, S. avenae* (Figure 1A). The highest GST activities were observed in *M. dirhodum* with significant difference compared to *R. padi* (2.51-fold) and *S. graminum* (2.47-fold) (Figure 1B). However, the P450s activity of *M. dirhodum* at the same level as *R. padi* and P450s activity in *S. graminum* was significantly lower than *S. avenae* (3.05-fold) (Figure 1C).

### Inducible Enzyme Activity

**FIGURE 2:**
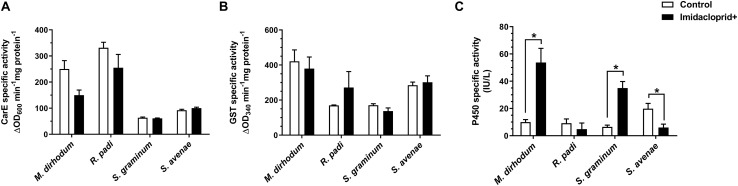
Effect of imidacloprid on detoxifying enzyme activities of carboxylesterase (CarE, **A**), glutathione S-transferase (GSTs, **B**), and cytochrome P450 monooxygenase (P450s, **C**) in four aphid species in wheat. Error bars represent the mean ± SEM (*n* = 3). Significant differences between imidacloprid treated and control samples were marked by asterisks: *significant at the 0.05 level.

Furthermore, we analyzed the detoxifying enzyme activities of four wheat aphids after imidacloprid treatment. The activities of P450s in *M. dirhodum* and *S. graminum* were significantly higher in imidacloprid-treated aphids than untreated by 5.37 and 5.39-fold (Figure 2C), respectively. However, the activity of P450s was significantly lower in the imidacloprid treated than control in *S. avenae*. The activities of CarE and GSTs were no difference between treated and untreated among four wheat aphids (Figures 2A,B).

### Synergism Bioassay

The effects of PBO, TPP, and DEM on imidacloprid activities against four wheat aphids was presented in Table 2. The susceptibilities of *M. dirhodum* and *S. graminum* to imidacloprid were increased by the oxidase inhibitor PBO (20.06 and 2.50-fold), which consistent with the enzyme activities induced by imidacloprid. The susceptibility of *R. padi* to imidacloprid was increased by TPP (6.88-fold) and DEM (8.32-fold). In contrast, TPP, DEM, and PBO had inconsequential effects on imidacloprid against *S. avenae.*

**TABLE 2:** Synergistic effects of PBO, DEM, and TPP on imidacloprid against four aphid species in wheat.

Species	Insecticides/insecticide + synergist	N^*a*^	Slope ± SE	*P-*value	LC_50_ (95% CI) ^*b*^mg L^–1^	SR^*c*^
*M. dirhodum*	Imidacloprid	887	0.83 ± 0.07	0.0013	261.91(177.09−387.35)	1.00
	imidacloprid + PBO	591	0.81 ± 0.04	0.0002	13.05(10.06−16.94)	20.06
	Imidacloprid + DEM	276	0.61 ± 0.16	0.1614	96.31(30.25−306.66)	2.72
	Imidacloprid + TPP	534	0.76 ± 0.15	0.0138	80.30(37.32−172.76)	3.26
*R. padi*	Imidacloprid	884	0.76 ± 0.19	0.0289	65.21(27.80−152.99)	1.00
	Imidacloprid + PBO	939	1.38 ± 0.16	0.0138	37.58(24.47−57.71)	1.74
	Imidacloprid + DEM	723	0.46 ± 0.05	0.0139	7.84(4.21−14.59)	8.32
	Imidacloprid + TPP	590	0.67 ± 0.01	0.0002	9.48(8.85−10.16)	6.88
*S. graminum*	Imidacloprid	873	0.50 ± 0.08	0.0088	41.11(23.84−70.89)	1.00
	Imidacloprid + PBO	455	1.16 ± 0.36	0.1921	16.46(3.36−80.69)	2.50
	Imidacloprid + DEM	327	0.66 ± 0.18	0.1713	41.78(13.63−128.11)	0.98
	Imidacloprid + TPP	453	0.64 ± 0.09	0.0854	39.70(22.81−69.11)	1.04
*S. avenae*	Imidacloprid	481	1.11 ± 0.08	0.0054	6.79(4.81−9.59)	1.00
	Imidacloprid + PBO	500	1.23 ± 0.11	0.0081	16.68(11.36−24.49)	0.41
	Imidacloprid + DEM	480	0.50 ± 0.07	0.0204	16.30(8.76−30.31)	0.42
	Imidacloprid + TPP	521	0.66 ± 0.08	0.0132	18.18(11.21−29.47)	0.37

*^*a*^The number of aphids tested.*

*^*b*^Median lethal concentration (LC_50_), 95% confidence interval (95% CI).*

*^*c*^The LC_50_ of insecticide/The LC_50_ of insecticide with synergist.*

## Discussion

Detecting the susceptibility of insecticide can help with the assessment when exponential growth is limited by insecticide-dependent factors (Mohammed et al., 2019). In this study, we assessed the tolerance of four wheat aphids to seven insecticides. The data revealed that most of the insecticides have much lower toxicity to *M. dirhodum* than other three wheat aphids. This means that *M. dirhodum* has the highest insecticide tolerance (especially for the neonicotinoids imidacloprid and thiamethoxam), whereas *R. padi* seems more susceptible to most of insecticides that we tested in this study. These results are consistent with the result that *M. dirhodum* has potential becoming a dominant aphid species in fields (Honek et al., 2006). Similarly, studies comparing the insecticide tolerances between *S. avenae* and *R. padi* also proved that most insecticides had lower toxicity to *S. avenae* (Lu and Gao, 2016).

Developing an appropriate pest control strategy should ideally consider the insecticide tolerance difference among pest species. *Bemisia tabaci*, an agriculturally important pest worldwide with B and Q two biotypes, different biological characteristics between biotypes B and Q, especially insecticide tolerance, affected the outcome of their competition (Yao et al., 2017). An earlier investigation confirmed that applications of either pyriproxyfen or neonicotinoids may select for biotype Q, enabling it to survive to a greater degree in areas where these insecticides are applied (Horowitz et al., 2005). Thus, *B. tabaci* biotype Q has supplanted biotype B as the major biotype in China, where it causes serious economic losses (Wang et al., 2010). The results of the current study suggest that *M. dirhodum* may replace *R. padi* and *S. avenae* become dominant species in wheat where imidacloprid and thiamethoxam used.

Insecticide-detoxifying enzymes are important for the metabolism of xenobiotics in insects. The resistance of insects to insecticides are relate to the increases of these detoxifying enzymes activities. Our study confirmed that four wheat aphids have different tolerance to insecticides. *Metopolophium dirhodum* with the highest tolerance to insecticide may relate to the activity of CarE significantly higher than that in *S. graminum* and *S. avenae*. Moreover, the highest activity of GSTs was also observed in *M. dirhodum*. Another study obtained similar result the authors examined the GSTs activities and molecular weights in *M. dirhodum*, *S. avenae* and *R. padi*, and determined that the highest GSTs active was observed in the extracts from *M. dirhodum* (Leszczynski et al., 1994). Previous research also indicated that the detoxification efficiency of GSTs is likely higher in *S. avenae* than *R. padi* (Lu and Gao, 2009), which is consistent with our results. Although *R. padi* seems more susceptible to most of insecticides, the activity of CarE at the same level as *M. dirhodum*, significantly higher than *S. avenae* and *S. graminum*.

The accumulation of P450 is the main mechanism underlying the imidacloprid resistance in *N. lugens* (Zhang et al., 2016a,c; Zimmer et al., 2018). But there was no significant difference between *M. dirhodum* and other three aphid species in P450 activities. We further determined the detoxifying enzyme activities of four wheat aphids with imidacloprid treated. The subsequent analysis showed greater P450 activities in *M. dirhodum* than untreated. Furthermore, the imidacloprid susceptibility was suppressed by PBO in *M. dirhodum* (20.06-fold). These results imply that the P450 activity induced by imidacloprid may be related to the insensitivity of *M. dirhodum* to imidacloprid. Expression induction of P450 genes by imidacloprid in *N. lugens* showed some P450 genes (*CYP6CS1*, *CYP6CW1*, and *CYP6ER1*) were up-regulated genes (Zhang et al., 2016a). CYP6 also have been reported to play important roles in imidacloprid resistance in *Drosophila melanogaster* (Daborn et al., 2001), *Bemisia tabaci* (Karunker et al., 2008), *Myzus persicae* (Puinean et al., 2010). The detoxifying enzyme activities in *R. padi* were not significantly affected by imidacloprid, but TPP significantly increased the imidacloprid effects (6.88-fold). Similarly, a previous study demonstrated that TPP suppressed imidacloprid activity by 2.45-fold in an imidacloprid-resistant *R. padi* strain (Wang et al., 2018) and another study have proved that GSTs can influences imidacloprid effect (Sillapawattana and Schaffer, 2017) suggesting that GSTs may related to susceptibility to imidacloprid in *R. padi*. Curiously, the activity of P450s in *S. avenae* with imidacloprid treated was significantly lower than control, both PBO and TPP show significant antagonism of imidacloprid in *S. avenae* which need further study, but PBO showed antagonism with some insecticides that have been reported (Ullah et al., 2017).

In conclusion, this study revealed the difference in susceptibilities of four aphid species in wheat to various insecticides. The insensitivity of *M. dirhodum* to imidacloprid may related to an inducible increase activity of P450.

## Data Availability Statement

The raw data supporting the conclusions of this article will be made available by the authors, without undue reservation, to any qualified researcher.

## Author Contributions

PG, DC, and XZ conceived and designed the research. PG, CW, and ML conducted the experiments. PG and XAL analyzed the data. PG and XZ wrote the manuscript. YZ and XRL revised the manuscript. All authors have read and approved the manuscript.

## Conflict of Interest

The authors declare that the research was conducted in the absence of any commercial or financial relationships that could be construed as a potential conflict of interest.
